# Cheetahs cannot fool biologgers

**DOI:** 10.1093/conphys/coy063

**Published:** 2018-12-20

**Authors:** Björn Illing

**Affiliations:** ARC Centre of Excellence for Coral Reef Studies, James Cook University, Townsville, QLD, Australia

Understanding the behavior of large, endangered and often iconic species in the wild is critical for assessing the success of conservation efforts. This used to require long hours of observation by scientists and volunteers. However, with the advent of technologies such as biologgers, studying animals in their natural habitat has become much more feasible. But what exactly is a biologger? You can think of a biologger like the black box in an airplane that records important information. This could include, for example, the position of the object or information about some of its important functions. In animals, biologgers can record vital functions, for example, body temperature, heart rates and/or movement. This information can be used to draw conclusions, for instance, on the study species’ biology and how it responds to environmental stress. Using biologgers, Robyn Hetem and colleagues (2018) used long-term body temperature and activity monitoring to understand how well cheetahs, previously kept in a rehabilitation program, would perform upon their release into the wild.

Cheetahs are known to be the fastest land animals in the wild, with burst speeds of 80–120 km/h during hunts. However, they can lose up to every tenth kill to stronger competitors, like lions, leopards or hyenas. With the biologgers, the team could associate peaks in cheetah body temperature with hunting success. They related the body temperature spike to the fear of getting attacked by competitors and losing the prey. So, by linking body temperature and activity data from the biologgers, the researchers could identify the number of hunts, success rates and the time at which the animals where active. What was also interesting was that, despite the immense seasonal differences in air temperatures—ranging from freezing in winter to 40°C in summer—cheetahs maintained nearly constant body temperatures (within 2°C) and their hunting success. The cheetahs hunted one–three times per day and had success every second or third attempt. Hunting was neither related to the time of day nor the heat they experienced, but rather to the time that the animals were active. The authors concluded that it was probably not too hot to hunt, but rather it was just too hot to be active in general. Contrary to previous beliefs that cheetahs would predominantly hunt during the daytime to avoid losing prey after successful hunts, Hetem and her team could show that 25% of the cheetahs’ general and hunting activity occurred when it was dark.

How do these findings support cheetah conservation? The animals used in this study were habituated to human presence and part of a rehabilitation program for injured or orphaned cheetahs in Namibia. Here, approximately half (about 3500 individuals) of the remaining wild population of this red-listed species can be found. Long-term monitoring programs for cheetahs after they are released back into the wild will help assess the effectiveness of rehabilitation and other conservation strategies. In fact, Hetem and her team believe that biologgers can make a huge contribution toward wildlife biology research and will be used more often in the future.

**Figure:**
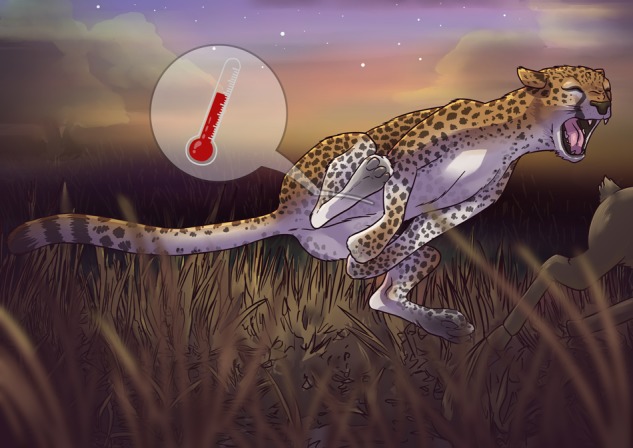


Illustration by Erin Walsh; Email: ewalsh.sci@gmail.com
